# Tracheotomy for the Removal of a Foreign Body

**Published:** 1856-04

**Authors:** M. G. Slaughter

**Affiliations:** Pinckneyville, Ala.


ATLANTA

Medical and Surgical Journal.

VOL. I.] APRIL, 1856. [NO. 8.

### ORIGINAL COMMUNICATIONS.

### ARTICLE I.

*Tracheotomy for the Removal of a Foreign Body.* By M. G. SLAUGHTER, M. D., Pinckneyville, Ala.

In August, 1854, I was called upon, to visit a patient, for the purpose of performing an operation, for the removal of water-melon seed from the Trachea. Owing to the peculiarity of my engagements, I could not immediately obey the summons, but promised to reach the patient as soon as possible, which I did, during the night. My patient was a little girl, about three years old, the daughter of Mr. A. D———. She was fleshy, with a very short neck. I found her in a fit of violent coughing, with a spasmodic difficulty of breathing; almost amounting to suffocation. These severe, and threatening paroxysm, however, would soon subside, but continued to return at short intervals. During the intervals, she seemed to rest well, and slept soundly. There was, however, a constant whistling, murmuring sound, and an entire loss of voice. I learned from the parents, that the child, with her mouth filled with water-melon, had attempted to run, and chanced to fall, and that these symptoms had come on immediately.

The usual remedies, such as exciting emesis, coughing, &c., had all been tried before I was summoned. For the want of proper assistants, and believing as I do, that the operation upon a subject of this kind (might I not add any kind?) is both difficult and dangerous, I determined to defer the performance of it until the morning, unless I should find that the life of my patient depended upon prompt and decided action. For, notwithstanding, the operation may appear very simple, to those whose only performance has been upon the dead subject—in a short-necked, fleshy child, or, even in the adult, it often proves very difficult. And, permit me to say, that the unexperienced Surgeon, however well educated, if not totally reckless of human life, who undertakes the operation, for the first time upon the living subject, will find difficulties which, perhaps, he never anticipated—he, judging of it alone, by his performances in the dissecting room. We know that some of the most experienced Surgeons who have ever lived, and whose skill and ability can never be doubted, or questioned, have been compelled to abandon, after commencing this operation, on account of unavoidable dangers.

Having procured such assistance as could be commanded, and prepared every thing necessary for the operation, the little patient was placed upon a table, on her back, the head thrown back. The patient being held in this position by assistants, I placed the fingers of my left hand upon the skin, near the median line, to steady it, and made an incision with the scalpel through the skin, directly upon the median line, extending from the inferior part of the larynx down to within a short space of the superior part of the sternum; raising the superficial fascia with the tenaculum, punctured, and divided it upon a director, then divided fat, and separated the sterno-thyroid muscle, at its junction. The trachea being exposed, I waited a short time for the hemorrhage to cease, which was soon accomplished by sponging with cold water, and then opened the trachea, incising four rings. Immediately two seed were expelled by the rush of air through the incision. Supposing all to have been removed, the wound was closed and dressed. We observed, however, after a short time, that although the violent cough, and the *spasmodic* difficulty of breathing, had subsided, yet the murmuring, whistling sound was still present. This, I supposed, to originate, either from some injury sustained by the lung and bronchiæ, from the seed having remained so long in the trachea, (about forty hours,)

producing these constant spells of spasmodic cough, or, that a seed still remained in one of the bronchi, and by some means firmly fixed there. Owing to this continued difficulty of breathing, and the fretfulness of the child, the wound did not heal by the first intention. One night, about a week after the operation, the difficulty of breathing suddenly subsided, and upon removing the dressing, next morning, a seed was found upon it—having been expelled through the wound. After this, the wound healed readily. Some slight inflammation of the lung, the result of the long continuance of the foreign body, yielded very kindly to appropriate treatment.

